# Normal Values and Patterns of Normality and Physiological Variability of Mitral and Tricuspid Inflow Pulsed Doppler in Healthy Children

**DOI:** 10.3390/healthcare10020355

**Published:** 2022-02-11

**Authors:** Massimiliano Cantinotti, Pietro Marchese, Marco Scalese, Eliana Franchi, Nadia Assanta, Martin Koestenberger, Jef Van den Eynde, Shelby Kutty, Raffaele Giordano

**Affiliations:** 1 Fondazione G. Monasterio CNR-Regione Toscana, 54100 Massa, Italy; cantinotti@ftgm.it (M.C.); pitrino91@gmail.com (P.M.); efranchi@ftgm.it (E.F.); assanta@ftgm.it (N.A.); 2 Institute of Clinical Physiology, 56121 Pisa, Italy; scalese@ftgm.it; 3 Department of Pediatrics, Division of Pediatric Cardiology, Medical University, 8036 Graz, Austria; martin.koestenberger@medunigraz.at; 4 Helen B. Taussig Heart Center, Johns Hopkins Hospital, Baltimore, MD 21218, USA; jvanden9@jhu.edu (J.V.d.E.); shelby.kutty@gmail.com (S.K.); 5 Adult and Pediatric Cardiac Surgery, Department of Advanced Biomedical Sciences, University of Naples “Federico II”, 80138 Naples, Italy

**Keywords:** diastolic function, doppler, heart defects, congenital, echocardiography, right ventricle, ventricular dysfunction

## Abstract

Background: While mitral (MV) and tricuspid valve (TV) pulsed Doppler velocities and derived gradients are commonly evaluated, data on normal pediatric values are limited. This study aimed to evaluate the normal values and physiological variability for MV and TV Doppler velocities and derived gradients in a large cohort of prospectively enrolled healthy children. Methods: The echocardiographic measurements included pulsed Doppler MV and TV E and A velocities, E deceleration times (EDT), maximal and mean gradients, and velocity time integral (VTI). Results: A total of 544 healthy subjects (median age 6.4 years, range 1 day–17.68 years) were included. MV and TV E velocity, E/A ratio, and E and A wave duration increased, while A velocity decreased with age (*p* < 0.001). Along with an increase in VTI, there occurred a progressive increase in maximum velocity and gradients and a decrease in mean velocities and gradients. E/A inversions were common, especially at the TV in neonates and infants. For MV, inversion in either one, two, or three consecutive beats occurred in 51.9% of neonates and 18.3% of infants, while it was rare at older ages (all *p* < 0.001). For TV, inversions in three consecutive beats occurred in 71.4% of neonates, while inversions in only one or two beats were more common in infants (27.3%). For TV, inversion in one or more beats, however, was not infrequent at all ages. Conclusions: We report normal values and patterns of normality and physiological variability for MV and TV inflow Doppler from a large population of healthy children.

## 1. Introduction

Cardiac inflow Doppler velocities and pressure gradients are sensitive echocardiographic indices of atrioventricular valve pathology and ventricular diastolic function [1,2]. However, few studies systematically quantifying pulsed wave Doppler velocities and pressure gradients have been reported in children, especially for the tricuspid valve (TV) [3,4]. Because of this limitation, pediatric cardiologists often apply adult nomograms clinically, which is inadequate.

The few published studies for mitral (MV) [5,6,7,8,9,10,11] Doppler velocities in children are limited by their relatively small sample size, and only a single study [11] assessed TV Doppler velocities. Other echocardiographic indices of inflow stenosis, including velocity time integrals (VTI) and Doppler-derived gradients, have not been studied [11,12,13,14,15,16,17]. Likewise, investigations into the age-dependent variation of Doppler inflow indices have been limited [5,6,7,8,9].

The aim of the present study was to establish reference values, patterns of normality, and physiological variability for pediatric MV and TV pulsed Doppler velocities and derived gradients in healthy children. 

## 2. Materials and Methods

### 2.1. Study Population

The study enrolled 554 healthy children (median age 6.4 years, range 1 day–17.68 years; 238 female) identified prospectively from referrals to the outpatient pediatric cardiology department at the Fondazione CNR-Regione Toscana G. Monasterio of Massa, from August 2017 to June 2019. Reasons for echocardiographic referral included primarily the presence of a cardiac murmur or an abnormal sports screening assessment. All subjects with clinical, electrocardiographic, or echocardiographic evidence of congenital or acquired heart disease were excluded from the healthy cohort. Small intracardiac defects, such as patent foramen ovale, were considered as normal findings [16]. Other exclusion criteria were: (i) known or suspected neuro-muscular disease, genetic syndromes, or chromosomal abnormalities; (ii) body mass index (BMI) ≥ 95th percentile for children ≥ 2 years old or weight-for-length Z-score ≥ 2 based on the World Health Organization (WHO) Child Growth Standards for children < 2 years old; (iii) pulmonary hypertension; (iv) systemic hypertension (for children > 4 year of age); (v) connective tissue disease; (vi) family history of genetic cardiac disease (such as Marfan syndrome or cardiomyopathy) [18]. Pre-term and/or low-weight neonates were also excluded. All patients underwent a complete 2-dimensional examination, and images were digitally stored for subsequent offline analysis. Images were collected only in quiet and cooperative children. No child was sedated. Approval for this study was obtained from the Local Ethics Committee (Study “Bet” N.390). Parents or legal guardians of all the children were informed and accepted to participate in the study by signing a written consent. 

### 2.2. Echocardiographic Examinations

Images were obtained in the standard left ventricle (LV) four-chamber view. The following parameters were evaluated on the offline analysis: pulsed Doppler velocities (cm/s), E maximal velocity (cm/s), A maximal velocity (cm/s), E duration (ms), A duration (ms), E deceleration time (EDT) (ms), VTI measurements (cm), and derived maximal and medium gradient (mmHg). The measurements were averaged across three consecutive beats. LV ejection fraction (LVEF) was calculated by the biplane Simpson’s method [19,20,21]. Global longitudinal strain (GLS) was calculated for both the LV and the RV, as described previously [18]. Measurements were only made if excellent and unambiguous views were available. Two experienced pediatric cardiologists (M.C. and E.F.) independently acquired images and performed the measurements.

### 2.3. Statistical Analyses

Continuous data were expressed as mean ± standard deviation (SD) or as median and IQR, as appropriate, and compared using Student’s *t*-test, ANOVA test, Mann–Whitney U test, or Kruskal–Wallis test, as appropriate. Categorical variables were expressed as *n* (%) and compared using the Chi-squared test. Bonferroni post hoc correction was used to account for multiple comparison. Normality of the data was assessed using Shapiro–Wilk and Kolmogorov–Smirnov tests. Variability in EDT as well as in E and A velocities values among different beats was analyzed based on the SD of 3 consecutive values.

The sample size for the present study was calculated based on previous observations [18]. To examine the relationship of each of the echocardiographic parameters with age, sex, weight, height, body surface area (BSA, based on Haycock formula), and heart rate (HR), various types of regression models (including linear, logarithmic, quadratic, cubic, power, exponential, and square-root equations) were fitted to the data. Among the models that satisfied the assumption of homoscedasticity, the model with the highest R^2^ value was considered to provide the best fit. The presence or absence of heteroscedasticity, a statistical term used to describe the behavior of variance and normality of the residuals, was tested using the White test and the Breusch–Pagan test. To test the normality of residuals, the Shapiro–Wilk and Kolmogorov–Smirnov tests were used. Outliers were assessed visually and using the Leverage values and the Studentized error residuals, and observations were omitted in the final analysis if they deviated significantly from the models. Inter- and intra-rater agreement of the measurements was based on the coefficient of variation (CV) and intraclass correlation coefficients (ICC) in 20 subjects. CV were calculated as an average value from individual CVs for all the duplicates. Inter-rater CVs of less than 15% are generally acceptable, while intra-rater CVs should be less than 10%. The Statistical Package for Social Sciences (SPSS) Release 23.0 (IBM, Chicago, IL, USA) and Stata Version 13 for Windows (Stata Corporation, College Station, TX, USA) were used for all analyses, and a *p*-value < 0.05 was considered significant.

## 3. Results

### 3.1. Study Population

The characteristics of the study population are summarized in Table 1. The healthy cohort comprised 554 subjects (age range, 1 day–17.68 years, median 6.4 years, interquartile range [IQR] 2.1–9.9 years; 238 female). The subjects were divided into groups based on age: (1) neonates, 0 to 30 days, (2) infants, 1 to 24 months, (3) toddlers, 2 to 5 years, (4) children, 5 to 11 years, and (5) adolescents, 11 to 18 years.

### 3.2. Feasibility

Not all the measurements were feasible in all patients. Feasibility, however, was generally high (varying from 98.0% to 99.3%).

### 3.3. Data Normalization

The measurements were modeled with height, weight, BSA, HR, and age. Regressions showed generally low coefficients of determination (R^2^) for all MV and TV flow indices (R^2^ = 0.001–0.363; Supplementary Material, Table S1). This hampered the ability to calculate the Z-scores with sufficient reliability. Therefore, the data are presented as means ± SD, stratified for age groups (Table 2) and percentile tables (Table 3). Skewness and kurtosis of measurements by age groups demonstrated different shapes of distribution for different parameters; however, a normal distribution could be found in most subgroups (Supplementary Material, Table S2).

### 3.4. Correlations of MV/TV Doppler Parameters with BSA, HR, and Age 

Significant correlations of most echocardiographic parameters with BSA, HR, and age were found (*p* < 0.01), with limited exceptions (Supplementary Material, Table S3). However, the correlations were mostly weak (r < 0.50). The strongest correlations with BSA, HR, and age were found for VTI and EDT, for both the MV and the TV. Despite these significant correlations, linear regression modeling showed only low R^2^ and significant dispersions, suggesting that these variables only explained a small amount of the observed variability. This was especially the case at lower ages and higher HR (Supplementary Material, Table S3). The inter-observer and intra-observer CV and ICC showed good reproducibility (Supplementary Material, Table S4).

### 3.5. Normal Values for MV/TV Velocities and Derived Gradients 

#### 3.5.1. Mitral Inflow

MV maximal velocity and maximal gradients were both lower in neonates (Group 1) than in all other age groups (Table 2). MV EDT, E velocity, E duration, and E/A ratio were lower in neonates and infants (Groups 1 and 2) compared with older children. MV VTI progressively increased with age (significant in all pairwise comparisons). Conversely, MV mean velocity and mean gradients were significantly higher in infants (Group 2) than in all other age groups (Table 2). Similarly, MV A velocity was higher in neonates and infants (Groups 1 and 2) compared with older children.

#### 3.5.2. Tricuspid Inflow

TV maximal velocity and maximal gradient were higher in infants (Group 2) compared with toddlers (Group 3), while no other significant differences were noted among other age groups for these parameters. TV VTI, EDT, E velocity, E duration, and E/A ratio were all lower in neonates and infants (Groups 1 and 2) compared with older children. Furthermore, TV A duration was shorter in Groups 1–3 compared with Groups 4 and 5. Conversely, TV mean velocity, mean gradient, and A velocity were all higher in neonates and infants (Groups 1 and 2) compared with older children.

### 3.6. E/A Pattern Variability in Healthy Subjects at Different Ages 

#### 3.6.1. Mitral Inflow

The occurrence of E/A inversion is summarized in Table 4. E/A inversion at the MV in either one, two, or three consecutive beats was significantly more common in neonates and infants (Groups 1 and 2) compared with older groups (all *p* < 0.001). E values were constantly lower than A in three consecutive beats in 29.6% of participants in Group 1 and 8.7% of participants in Group 2. In contrast, E/A inversion was rare in children older than 2 years of age (Group 3–5, Table 4). 

#### 3.6.2. Tricuspid Inflow

E/A inversion at the TV was more commonly observed than in the MV, with 71.4% of children in Group 1 and 38.4% of children in Group 2 demonstrating inversion in all three consecutive beats, which was significantly different between these groups and significantly more frequent than in all other age groups. On the other hand, inversions in only one or two beats were less common in neonates (Group 1), although more common in infants (Group 2) compared with children older than 5 years of age (Groups 4 and 5, Table 4).

## 4. Discussion

We herein report pediatric normative data for MV and TV inflow Doppler flow indices in the largest homogeneous cohort of healthy children to date. This is also the first study in children to comprehensively evaluate a full set of MV and TV Doppler indices. Previous reports were limited to single [8,9,10] or few indices [7], and VTI and Doppler-derived gradients were not investigated previously. We further evaluated physiological variations of these indices through additional time acquisitions. 

The data presented here cover a gap in present pediatric nomograms of MV and TV Doppler indices. As reported for other echocardiographic indices [18], there exist important age-related differences with inflow Doppler assessments. In this study, the MV and TV E and A velocities changed primarily during the infant and neonatal period, with values that stabilized after 2 years of age. We demonstrated that E velocity, E/A ratio, and E and A wave duration increased, while A velocity decreased. These data are in agreement with previously published data on MV [5,11] and TV [6,7,10,14,15,16,17] Doppler patterns. Mechanistically, these observations are linked to the adaptive changes in diastolic dysfunction that occur soon after birth during the transition from the fetal to the neonatal environment [22]. During this time, sudden hemodynamic changes induce changes in titin isoform expression and passive stiffness [23].

As expected, a progressive increase in VTI was observed at both the MV and the TV, correlating with the increasing stroke volumes in a growing heart. Interestingly, there occurred a simultaneous progressive increase in maximum velocity and gradients at the MV, but not at the TV. With regard to mean velocities and gradients, those at the MV transiently increased in infancy and then returned to neonatal levels, whereas those at the TV decreased below neonatal and infant levels at older ages. These findings suggest that differences between both valves are present in terms of the maximum and the mean velocities and gradients during development. Of note, whereas the MV is ellipsoid-shaped, the TV orifice is distorted, which may predispose it to a transiently higher resistance to flow in conjunction with the increased ventricular stiffness observed in neonates and infants [24].

As demonstrated in this study, E/A inversions were frequent in healthy children, especially at lower ages (inversion in three consecutive beats in more than half of all neonates) and at the TV. In contrast, these were practically not observed at the MV after the age of 2 years. Physiological variations of TV inflow Doppler velocities during consecutive beats have previously been described [12,13,14,15] and have generally been attributed to acquisition of Doppler data in different phases of the respiratory cycle. Previous adult studies [12,13,14] showed that during inspiration, the MV E velocity mildly decreased, corresponding to decreased LV filling [8,9], while TV peak E velocity frankly increased, corresponding to increased systemic venous return [9,12,13]. Data on Doppler variability among different phases of the cardiac cycle in children are exceedingly limited but demonstrate that a similar phenomenon is also present in young children [15]. Accordingly, our data show variability of E/A patterns, especially for the TV, that seems to reflect physiological heart rate variability (that may be considered as a covariate of the respiratory frequency). Thus, the high prevalence of E/A inversions among healthy subjects should be considered mostly physiological, as a reflection of respiratory and heart rate variability. While E/A inversion typically represents diastolic dysfunction in adults [22], they may be related to the normal maturation of valve structures and ventricular diastolic function in children. Therefore, care should be taken not to apply adult criteria and cut-offs for atrioventricular valve disease in the pediatric population, especially in neonates and infants. In this regard, the present study provides important benchmark data to determine normal variability in clinical practice.

### Limitations

A complete set of measurements was not available for all studied subjects. We confirmed the difficulties to normalize pediatric Doppler indices by age, BSA, or other body size variables [5]; thus, we were limited to present data as mean values and percentiles. Finally, the population was constituted only of Caucasian children; thus, data from different ethnic backgrounds are lacking. The influence of confounders, including race and ethnic groups, needs to be examined in future studies.

## 5. Conclusions

We herein report normal values for Doppler flow parameters at the atrioventricular valves in a large population of healthy children. This is the first pediatric study to report a comprehensive dataset of normal values for Doppler velocities, derived gradients, and VTI values. We demonstrated physiological variability in E/A patterns that may occur especially at lower age and for the TV. These data may serve as a benchmark to determine normal variability in clinical practice and to support further studies on diastolic patterns in children with congenital and acquired cardiac defects.

## Figures and Tables

**Table 1:** Study population.

Variable	Male (*N* = 316)	Female (*N* = 238)	Total (*N* = 554)
Age, months	77.4 (0–212.14)	73.2 (0–211.54)	76.5 (0–212.14)
Age group			
0–30 days	*N* = 15	*N* = 13	*N* = 28
1–24 months	*N* = 54	*N* = 52	*N* = 106
2–5 years	*N* = 50	*N* = 32	*N* = 82
5–11 years	*N* = 145	*N* = 93	*N* = 238
11–18 years	*N* = 52	*N* = 48	*N* = 100
Weight, kg	25.9 ± 16.2	24.4 ± 16.9	25.3 ± 16.5
Height, cm	115.2 ± 33.4	110 ± 36.6	113 ± 34.9
BSA, m^2^	0.90 ± 0.40	0.85 ± 0.43	0.88 ± 0.42
HR, bpm	88.4 ± 21	96.6 ± 24.1	91.8 ± 22.7
SAP, mmHg	106.1 ± 10.7	106.31 ± 12.9	106.2 ± 11.6
DAP, mmHg	60.6 ± 8.7	62.5 ± 11.9	61.4 ± 10.1
EF, %	67.54 ± 8.25	64.88 ± 9.3	66.56 ± 8.7
FAC, %	38.80 ± 6.57	40.00 ± 6.24	39.20 ± 6.4
LV GLS, -%	25.04 ± 4.3	23.58 ± 5.1	24.51 ± 3.7
RV GLS, -%	20.41 ± 6.26	23.98 ± 7.15	21.68 ± 7.2

Values are expressed as mean ± standard deviation. Age is expressed as median (range). B.p.m, beats per minute; BSA, body surface area; DAP, diastolic arterial pressure; CHD, congenital heart disease; FAC, fractional area change; GLS, global longitudinal change; HR, heart rate; LV left ventricle; RV, right ventricle; SAP, systolic arterial pressure.

**Table 2:** Mitral (MV) and tricuspid valve (TV) Doppler values in healthy subjects: population means and standard deviation of the measurements by age groups. EDT, E wave deceleration time; VTI, velocity time integral.

Measurements	Group 1: 0–30 Days	Group 2: 1–24 Months	Group 3: 2–5 Years	Group 4: 5–11 Years	Group 5: 11–18 Years	Total	Overall *p*-Value	Pairwise Comparisons
MV max velocity (cm/s)	0.71 ± 0.19	0.93 ± 0.13	0.99 ± 0.12	0.99 ± 0.14	0.99 ± 0.14	0.96 ± 0.15	<0.001	1 vs. all other; 2 vs. 3,4
MV mean velocity (cm/s)	0.41 ± 0.10	0.51 ± 0.09	0.46 ± 0.08	0.43 ± 0.10	0.44 ± 0.10	0.45 ± 0.10	<0.001	2 vs. all other
MV max gradient (mmHg)	2.18 ± 1.30	3.57 ± 1.02	4.01 ± 0.98	3.99 ± 1.19	3.97 ± 1.11	3.82 ± 1.19	<0.001	1 vs. all other; 2 vs. 4
MV mean gradient (mmHg)	0.73 ± 0.37	1.10 ± 0.39	0.90 ± 0.33	0.79 ± 0.35	0.83 ± 0.37	0.87 ± 0.38	<0.001	2 vs. all other
MV VTI (cm)	10.16 ± 3.59	13.5 ± 3.19	15.75 ± 2.88	17.17 ± 3.17	18.55 ± 3.59	16.15 ± 3.88	<0.001	all vs. all other
MV E velocity (cm/s)	0.66 ± 0.20	0.93 ± 0.16	1.00 ± 0.13	1.00 ± 0.15	1.00 ± 0.14	0.97 ± 0.17	<0.001	1,2 vs. all other
MV A velocity (cm/s)	0.63 ± 0.18	0.71 ± 0.14	0.59 ± 0.1	0.56 ± 0.12	0.58 ± 0.12	0.6 ± 0.14	<0.001	1 vs. 4; 2 vs. 3,4,5
MV E/A ratio	1.17 ± 0.63	1.35 ± 0.34	1.77 ± 0.4	1.85 ± 0.44	1.8 ± 0.42	1.7 ± 0.48	<0.001	1,2 vs. all other
MV EDT (msec)	88.1 ± 23.2	100.7 ± 23.4	125.2 ± 31.1	134.9 ± 30.9	148.8 ± 34.7	127.1 ± 34.9	<0.001	1,2 vs. all other; 3,4 vs. 5
MV E wave duration (msec)	127.6 ± 29.8	143 ± 30.1	171.6 ± 32.9	186.1 ± 29.3	203.1 ± 34.7	176 ± 38.2	<0.001	1,2 vs. all other
MV A wave duration (msec)	94.9 ± 18.7	96.5 ± 20.6	111.4 ± 21.6	121.2 ± 20.1	126.3 ± 23.5	114.7 ± 23.8	<0.001	1 vs. 2,3; 4 vs. 5
TV max velocity (cm/s)	0.67 ± 0.11	0.69 ± 0.13	0.64 ± 0.09	0.66 ± 0.1	0.68 ± 0.1	0.67 ± 0.11	0.019	2 vs. 3
TV mean velocity (cm/s)	0.38 ± 0.08	0.4 ± 0.08	0.34 ± 0.06	0.34 ± 0.06	0.36 ± 0.07	0.35 ± 0.07	<0.001	1,2 vs. 3,4; 2 vs. 5
TV max gradient (mmHg)	1.82 ± 0.58	2 ± 0.75	1.71 ± 0.5	1.81 ± 0.57	1.93 ± 0.56	1.85 ± 0.60	0.010	2 vs. 3
TV mean gradient (mmHg)	0.6 ± 0.25	0.66 ± 0.29	0.49 ± 0.17	0.48 ± 0.18	0.54 ± 0.21	0.53 ± 0.22	<0.001	1,2 vs. 3,4; 2 vs. 5
TV VTI (cm)	9.88 ± 3.6	11.18 ± 3.24	12.98 ± 3.27	14.5 ± 3.22	16.13 ± 3.21	13.71 ± 3.71	<0.001	1,2 vs. all other
TV E velocity (cm/s)	0.58 ± 0.39	0.60 ± 0.14	0.64 ± 0.09	0.66 ± 0.11	0.69 ± 0.11	0.65 ± 0.14	<0.001	1 vs. 3; 1,2 vs. 4,5
TV A velocity (cm/s)	0.62 ± 0.13	0.61 ± 0.16	0.46 ± 0.12	0.44 ± 0.11	0.47 ± 0.11	0.49 ± 0.14	<0.001	1,2 vs. all other
TV E/A ratio	1.12 ± 1.5	1.08 ± 0.42	1.49 ± 0.42	1.61 ± 0.43	1.56 ± 0.4	1.46 ± 0.57	<0.001	1,2 vs. all other
TV EDT (msec)	86.7 ± 29.1	97.8 ± 31.5	139.4 ± 40.2	155.4 ± 42.1	169.6 ± 47.7	141.5 ± 48.9	<0.001	1,2 vs. all other
TV E wave duration (msec)	130.9 ± 40	150.6 ± 45.5	201.5 ± 50.3	222 ± 45.8	239.2 ± 48.4	204.7 ± 57.6	<0.001	1,2 vs. all other
TV A wave duration (msec)	109.3 ± 25	117.9 ± 31.6	124 ± 26.2	136.7 ± 27.6	140.1 ± 29.2	130.6 ± 29.8	<0.001	1,2,3 vs. 4,5

EDT, E wave deceleration time; VTI, velocity time integral.

**Table 3:** Mitral (MV) and tricuspid valve (TV) Doppler values in healthy subjects: percentile measurements per age group.

Measurements	1–24 Months				2–5 Years				5–11 Years				11–18 Years			
	5th	10th	90th	95th	5th	10th	90th	95th	5th	10th	90th	95th	5th	10th	90th	95th
MV max velocity (cm/s)	0.74	0.79	1.12	1.16	0.83	0.85	1.17	1.21	0.77	0.82	1.18	1.27	0.76	0.80	1.15	1.21
MV mean velocity (cm/s)	0.38	0.41	0.61	0.69	0.33	0.36	0.57	0.60	0.28	0.31	0.56	0.60	0.29	0.31	0.55	0.61
MV max gradient (mmHg)	2.21	2.50	5.02	5.46	2.76	2.91	5.51	5.86	2.35	2.69	5.58	6.42	2.34	2.58	5.32	5.86
MV mean gradient (mmHg)	0.58	0.69	1.49	1.91	0.45	0.53	1.34	1.42	0.32	0.38	1.24	1.42	0.34	0.39	1.23	1.49
MV VTI (cm)	8.79	9.54	17.10	19.67	11.63	12.77	19.43	20.17	12.30	13.13	21.53	23.03	12.43	14.60	22.67	23.87
MV E velocity (cm/s)	0.67	0.73	1.14	1.22	0.84	0.86	1.18	1.24	0.77	0.82	1.20	1.27	0.77	0.81	1.17	1.22
MV A velocity (cm/s)	0.50	0.54	0.87	0.97	0.44	0.46	0.73	0.76	0.41	0.43	0.72	0.78	0.41	0.42	0.72	0.78
MV E/A ratio	0.84	0.99	1.70	1.94	1.25	1.34	2.24	2.45	1.29	1.36	2.41	2.66	1.31	1.37	2.43	2.56
MV EDT (msec)	67.7	73.0	129.3	144.3	76.3	93.0	158.3	182.0	83.7	98.7	172.7	180.3	96.1	108.2	195.5	204.3
MV E wave duration (msec)	105.7	109.7	186.3	209.0	128.3	137.3	212.5	226.7	140.0	152.3	223.3	231.3	137.3	167.7	237.5	261.2
MV A wave duration (msec)	69.0	72.3	122.7	129.3	83.7	87.3	137.3	151.0	93.5	97.7	144.7	163.0	88.8	98.2	153.5	167.0
TV max velocity (cm/s)	0.52	0.55	0.85	0.92	0.51	0.53	0.76	0.81	0.49	0.53	0.81	0.83	0.55	0.57	0.81	0.84
TV mean velocity (cm/s)	0.29	0.31	0.50	0.53	0.26	0.27	0.42	0.44	0.24	0.26	0.42	0.47	0.24	0.28	0.45	0.47
TV max gradient (mmHg)	1.10	1.19	2.89	3.40	1.05	1.11	2.34	2.61	0.99	1.12	2.63	2.85	1.21	1.28	2.64	2.90
TV mean gradient (mmHg)	0.33	0.40	1.02	1.13	0.27	0.30	0.73	0.78	0.23	0.27	0.71	0.87	0.23	0.30	0.81	0.89
TV VTI (cm)	7.59	8.01	15.85	19.80	8.69	9.45	17.70	18.80	9.47	10.22	18.67	20.30	11.21	11.95	20.07	21.17
TV E velocity (cm/s)	0.41	0.44	0.80	0.88	0.50	0.54	0.76	0.79	0.49	0.52	0.81	0.84	0.51	0.55	0.82	0.86
TV A velocity (cm/s)	0.38	0.44	0.83	0.86	0.33	0.34	0.61	0.69	0.30	0.32	0.57	0.64	0.33	0.34	0.59	0.69
TV E/A ratio	0.65	0.68	1.65	1.82	0.89	0.92	2.12	2.25	0.99	1.13	2.12	2.43	0.87	1.12	2.05	2.35
TV EDT (msec)	60.7	69.0	136.0	156.0	78.7	89.7	176.0	208.0	83.7	103.7	203.0	221.0	71.0	119.3	232.3	251.0
TV E wave duration (msec)	95.0	105.7	217.0	248.0	129.3	143.3	260.0	269.0	139.0	162.0	282.0	297.0	143.7	185.0	298.0	316.7
TV A wave duration (msec)	80.3	84.5	153.0	178.0	93.3	97.0	163.0	183.0	99.7	106.0	170.7	190.3	95.0	109.3	177.0	213.0

EDT, E wave deceleration time; VTI, velocity time integral.

**Table 4:** Mitral (MV) and tricuspid valve (TV) inflow E/A inversion in healthy subjects.

Measurements	Group 1: 0–30 Days	Group 2: 1–24 Months	Group 3: 2–5 Years	Group 4: 5–11 Years	Group 5: 11–18 Years	Total	Overall *p*-Value	Pairwise Comparisons
MV healthy subjects								
No inversion	13 (48.1)	85 (81.7)	79 (97.5)	233 (98.3)	100 (100)	510 (92.9)	<0.001	1,2 vs. all other
Inversion in 1 beat	3 (11.1)	6 (5.8)	1 (1.2)	2 (0.8)	0 (0)	12 (2.2)	<0.001	1 vs. 3; 1,2 vs. 4,5
Inversion in 2 beats	3 (11.1)	4 (3.8)	0 (0)	1 (0.4)	0 (0)	8 (1.5)	<0.001	1 vs. 3; 1,2 vs. 4,5
Inversion in 3 beats	8 (29.6)	9 (8.7)	1 (1.2)	1 (0.4)	0 (0)	19 (3.5)	<0.001	1 vs. 3; 1,2 vs. 4,5
TV healthy subjects								
No inversion	5 (17.9)	30 (30.3)	66 (80.5)	208 (87.8)	89 (89)	398 (72.9)	<0.001	1,2 vs. all other
Inversion in 1 beat	1 (3.6)	17 (17.2)	6 (7.3)	15 (6.3)	5 (5)	44 (8.1)	<0.001	2 vs. 4,5
Inversion in 2 beats	2 (7.1)	14 (14.1)	6 (7.3)	5 (2.1)	2 (2)	29 (5.3)	<0.001	2 vs. 4,5
Inversion in 3 beats	20 (71.4)	38 (38.4)	4 (4.9)	9 (3.8)	4 (4)	75 (13.7)	<0.001	1,2 vs. all other

## Data Availability

The data presented in this study are available on request from the corresponding author.

## Supplementary Materials

The following supporting information can be downloaded at: https://www.mdpi.com/article/10.3390/healthcare10020355/s1, Table S1: Mitral (MV) and tricuspid valve (TV) Doppler values in healthy subjects: linear regression models using body surface area (BSA, based on Haycock formula as an independent variables). The table shows the beta coefficients (B), standard error (SE), coefficient of determination (R2), normality tests (Shapiro–Wilk [SW] and Kolmogorov–Smirnov [KS] tests), and heteroscedasticity tests (Breusch–Pagan [BP] and White [W] tests). EDT, E wave deceleration time; VTI, velocity time integral; Table S2: Mitral (MV) and tricuspid valve (TV) Doppler values in healthy subjects: skewness (S) and kurtosis (K) per age group. EDT, E wave deceleration time; VTI, velocity time integral; Table S3: Mitral (MV) and tricuspid valve (TV) Doppler values in healthy subjects: Pearson correlations. BSA, body surface area; EDT, E wave deceleration time; HR, heart rate; VTI, velocity time integral. ** Correlation is significant at the 0.01 level (2-tailed). * Correlation is significant at the 0.05 level (2-tailed); Table S4: Inter- and intra-observer reliability analysis. CV, coefficient of variation; EDT, E wave deceleration time; ICC, intraclass correlation coefficient; VTI, velocity time integral.
