# Supplementary material for: Normal Values and Patterns of Normality and Physiological Variability of Mitral and Tricuspid Inflow Pulsed Doppler in Healthy Children
Source: Healthcare (Basel). 2022 Feb 11;10(2):355. doi: 10.3390/healthcare10020355 (PMC8871925; doi:10.3390/healthcare10020355)
Supplement: Supplementary file 1 [file healthcare-10-00355-s001.zip › healthcare-1550857-supplementary.pdf]

Supplementary Material

Table S1. Mitral (MV) and tricuspid valve (TV) Doppler values in healthy subjects: linear regression models using body surface area (BSA, based on Haycock formula as an independent variables).

Measurement	Intercept	B	SE	R^2	SW	KS	BP	W
MV max velocity (cm/s)	0.902	0.077	0.144	0.043	<0.001	<0.001	<0.001	<0.001
MV mean velocity (cm/s)	0.496	−0.046	0.098	0.034	<0.001	<0.001	<0.001	<0.001
MV max gradient (mmHg)	3.355	0.569	1.146	0.038	<0.001	<0.001	<0.001	<0.001
MV mean gradient (mmHg)	1.024	−0.171	0.371	0.033	<0.001	<0.001	<0.001	<0.001
MV VTI (cm)	11.704	5.169	3.146	0.310	<0.001	<0.001	<0.001	<0.001
MV E velocity (cm/s)	0.899	0.095	0.214	0.030	<0.001	<0.001	<0.001	<0.001
MV A velocity (cm/s)	0.683	−0.093	0.131	0.076	<0.001	<0.001	<0.001	<0.001
MV E/A ratio	1.345	0.411	0.449	0.127	<0.001	<0.001	<0.001	<0.001
MV EDT (msec)	89.937	42.763	29.974	0.253	<0.001	<0.001	<0.001	<0.001
MV E wave duration (msec)	127.577	55.595	30.091	0.363	<0.001	<0.001	<0.001	<0.001
MV A wave duration (msec)	92.299	25.675	21.283	0.195	<0.001	<0.001	<0.001	<0.001
TV max velocity (cm/s)	0.667	0.005	0.107	0.001	<0.001	<0.001	<0.001	<0.001
TV mean velocity (cm/s)	0.380	−0.027	0.071	0.022	<0.001	<0.001	<0.001	<0.001
TV max gradient (mmHg)	1.856	0.006	0.600	0.001	<0.001	<0.001	<0.001	<0.001
TV mean gradient (mmHg)	0.612	−0.090	0.220	0.025	<0.001	<0.001	<0.001	<0.001
TV VTI (cm)	9.592	4.743	3.124	0.276	<0.001	<0.001	<0.001	<0.001
TV E velocity (cm/s)	0.572	0.087	0.116	0.084	<0.001	<0.001	<0.001	<0.001
TV A velocity (cm/s)	0.588	−0.117	0.131	0.117	<0.001	<0.001	<0.001	<0.001
TV E/A ratio	1.105	0.411	0.540	0.091	<0.001	<0.001	<0.001	<0.001
TV EDT (msec)	90.783	58.666	41.760	0.246	<0.001	<0.001	<0.001	<0.001
TV E wave duration (msec)	137.176	77.668	47.082	0.311	<0.001	<0.001	<0.001	<0.001
TV A wave duration (msec)	108.255	25.445	27.950	0.120	<0.001	<0.001	<0.001	<0.001

Note: Table shows the beta coefficients (B), standard error (SE), the coefficient of determination (R^2), normality tests (Shapiro-Wilk [SW] and Kolmogorov-Smirnov [KS] tests), and

heteroscedasticity tests (Breusch-Pagan [BP] and White [W] tests). EDT, E wave deceleration time; VTI, velocity time integral.

Table S2. Mitral (MV) and tricuspid valve (TV) Doppler values in healthy subjects: skewness (S) and kurtosis (K) per age group.

Measurements	1-24 months			2-5 years			5-11 years			11-18 years		
	S	K	P-value	S	K	P-value	S	K	P-value	S	K	P-value
MV max velocity (cm/s)	0.53	0.26	0.035	0.62	−0.24	0.027	0.57	0.05	0.002	−0.05	−0.20	0.123
MV mean velocity (cm/s)	0.41	1.50	0.016	0.28	0.59	0.200	0.20	−0.30	0.200	0.39	0.75	0.200
MV max gradient (mmHg)	0.90	0.79	0.000	0.86	0.22	0.002	0.94	0.71	0.000	0.33	−0.01	0.200
MV mean gradient (mmHg)	1.22	2.74	0.001	0.92	1.78	0.050	0.74	0.44	0.002	1.31	4.30	0.049
MV VTI (cm)	0.71	0.98	0.042	1.00	2.04	0.173	0.19	−0.16	0.200	−0.30	0.00	0.200
MV E velocity (cm/s)	0.07	0.87	0.003	0.16	0.73	0.008	0.40	0.07	0.000	−0.01	−0.20	0.200
MV A velocity (cm/s)	0.42	0.82	0.200	0.47	0.09	0.200	0.76	0.91	0.004	0.55	1.07	0.200
MV E/A ratio	0.78	1.81	0.041	1.05	3.63	0.200	0.83	1.82	0.019	0.94	0.53	0.000
MV EDT (msec)	0.61	0.45	0.159	0.33	0.89	0.200	−0.06	0.49	0.200	−0.33	1.50	0.101
MV E wave duration (msec)	0.94	1.13	0.016	0.26	1.25	0.200	0.15	0.51	0.200	0.07	1.87	0.056
MV A wave duration (msec)	0.72	1.17	0.116	0.67	0.30	0.043	0.79	1.40	0.027	0.20	0.09	0.200
TV max velocity (cm/s)	0.48	0.69	0.200	0.39	−0.11	0.163	0.24	−0.02	0.023	0.25	−0.36	0.096
TV mean velocity (cm/s)	0.95	3.17	0.001	0.55	0.27	0.006	0.37	−0.17	0.023	0.19	0.18	0.200
TV max gradient (mmHg)	1.12	2.08	0.009	0.73	0.44	0.034	0.68	0.57	0.000	0.58	0.06	0.033
TV mean gradient (mmHg)	2.13	8.38	0.000	1.04	1.59	0.002	0.82	0.43	0.000	0.81	1.25	0.080
TV VTI (cm)	1.51	2.28	0.000	1.03	1.26	0.035	0.21	−0.39	0.200	0.23	−0.03	0.200
TV E velocity (cm/s)	0.69	0.20	0.002	0.03	0.03	0.200	0.39	0.73	0.200	−0.03	−0.42	0.200

TV A velocity (cm/s)	0.33	0.48	0.200	1.44	2.68	0.001	1.33	2.66	0.000	1.02	1.12	0.014
TV E/A ratio	1.32	2.11	0.000	0.38	−0.09	0.200	0.54	0.81	0.084	0.29	0.31	0.177
TV EDT (msec)	1.99	7.16	0.000	0.35	1.59	0.042	−0.08	0.50	0.200	−0.04	0.76	0.067
TV E wave duration (msec)	1.62	3.42	0.000	0.64	1.31	0.200	−0.16	0.12	0.200	−0.15	0.85	0.148
TV A wave duration (msec)	1.84	5.33	0.001	1.00	0.89	0.002	1.19	2.34	0.000	0.72	0.99	0.009

Note: EDT, E wave deceleration time; VTI, velocity time integral.

Table S3. Mitral (MV) and tricuspid valve (TV) Doppler values in healthy subjects: Pearson correlations

Measurements	Mean HR	BSA	Age
MV max velocity (cm/s)	−0.241 **	0.217 **	0.183 **
MV mean velocity (cm/s)	0.376 **	−0.190 **	−0.213 **
MV max gradient (mmHg)	−0.231 **	0.203 **	0.170 **
MV mean gradient (mmHg)	0.355 **	−0.185 **	−0.207 **
MV VTI (cm)	−0.479 **	0.560 **	0.512 **
MV E velocity (cm/s)	−0.262 **	0.252 **	0.217 **
MV A velocity (cm/s)	0.410 **	−0.279 **	−0.306 **
MV E/A ratio	−0.487 **	0.356 **	0.360 **
MV EDT (msec)	−0.468 **	0.506 **	0.493 **
MV E wave duration (msec)	−0.561 **	0.606 **	0.572 **
MV A wave duration (msec)	−0.456 **	0.445 **	0.437 **
TV max velocity (cm/s)	0.112 *	0.021	0.005
TV mean velocity (cm/s)	0.407 **	−0.154 **	−0.164 **
TV max gradient (mmHg)	0.126 **	0.007	−0.006
TV mean gradient (mmHg)	0.412 **	−0.164 **	−0.171 **
TV VTI (cm)	−0.422 **	0.530 **	0.489 **
TV E velocity (cm/s)	−0.196 **	0.298 **	0.261 **
TV A velocity (cm/s)	0.544 **	−0.344 **	−0.334 **
TV E/A ratio	−0.504 **	0.301 **	0.285 **
TV EDT (msec)	−0.534 **	0.500 **	0.501 **
TV E wave duration (msec)	−0.600 **	0.561 **	0.534 **

TV A wave duration (msec)	−0.347 **	0.352 **	0.319 **

Note: BSA, body surface area; EDT, E wave deceleration time; HR, heart rate; VTI, velocity time integral. ** Correlation is significant at the 0.01 level (2-tailed). * Correlation is significant at the 0.05 level (2-tailed).

Table S4. Inter- and intra-observer reliability analysis.

Measurements	*ICC Inter-observer*	*ICC Intra-observer*	*CV Inter-observer*	*CV Intra-observer*
MV max velocity (cm/s)	0.775 (0.628-0.899)	0.760 (0.622-0.849)	5.3	3.2
MV mean velocity (cm/s)	0.769 (0.631-0.850)	0.763 (0.655-0.845)	5.3	3.3
MV max gradient (mmHg)	0.907 (0.789-0.960)	0.812 (0.639-0.860)	4.5	4.8
MV mean gradient (mmHg)	0.914 (0.793-0.947)	0.814 (0.781-0.937)	9.7	7.5
MV VTI (cm)	0.77 (0.628-0.819)	0.836 (0.648-0.889)	5.4	3.3
MV E velocity (cm/s)	0.91 (0.789-0.963)	0.932 (0.780-0.973)	10.7	7.7
MV A velocity (cm/s)	0.914 (0.798-0.947)	0.737 (0.638-0.851)	5.5	3.4
MV EDT (msec)	0.745 (0.628-0.799)	0.932 (0.882-0.974)	11.4	7.8
MV E wave duration (msec)	0.921 (0.869-0.964)	0.929 (0.79-0.970)	5.2	2.1
MV A wave duration (msec)	0.918 (0.792 -0.959)	0.938 (0.782-0.951)	4.3	2.1
TV max velocity (cm/s)	0.83 (0.798-0.91)	0.811 (0.698-0.861)	12.9	7.6
TV mean velocity (cm/s)	0.914 (0.758-0.954)	0.824 (0.738-0.941)	4.5	3.9
TV max gradient (mmHg)	0.732 (0.58-0.899)	0.72 (0.58-0.799)	12.8	7.5
TV mean gradient (mmHg)	0.907 (0.789-0.960)	0.77 (0.689-0.860)	9.3	6.9
TV VTI (cm)	0.905 (0.786-0.960)	0.896(0.865-0.975	10.6	7.6
TV E velocity (cm/s)	0.924 (0.858-0.944)	0.742 (0.618-0.791)	6.5	3.4
TV A velocity (cm/s)	0.912 (0.798-0.947)	0.77 (0.638-0.851)	5.1	6.4
TV EDT (msec)	0.84 (0.628-0.959)	0.93 (0.882-0.974)	7.4	7.8
TV E wave duration (msec)	0.893 (0.86-0.965)	0.912 (0.89-0.97)	5.2	9.2
TV A wave duration (msec)	0.911 (0.792 -0.95)	0.921 (0.792-0.951)	8.2	5.1

Note: CV, coefficient of variation; EDT, E wave deceleration time; ICC, intraclass correlation coefficient; VTI, velocity time integral.
